# Trends in diabetic ketoacidosis‐ and hyperosmolar hyperglycemic state‐related mortality during the COVID‐19 pandemic in the United States: A population‐based study

**DOI:** 10.1111/1753-0407.13591

**Published:** 2024-08-13

**Authors:** Xinyuan He, Amy Huaishiuan Huang, Fan Lv, Xu Gao, Yuxin Guo, Yishan Liu, Xiaoqin Hu, Jingyi Xie, Ning Gao, Yang Jiao, Yuan Wang, Jian Zu, Lei Zhang, Fanpu Ji, Yee Hui Yeo

**Affiliations:** ^1^ Department of Infectious Diseases The Second Affiliated Hospital of Xi'an Jiaotong University Xi'an China; ^2^ Department of Internal Medicine University of Connecticut School of Medicine Farmington Connecticut USA; ^3^ School of Mathematics and Statistics Xi'an Jiaotong University Xi'an China; ^4^ Division of Gastroenterology The Second Affiliated Hospital of Xi'an Jiaotong University Xi'an China; ^5^ Department of Endocrinology The Second Affiliated Hospital of Xi'an Jiaotong University Xi'an China; ^6^ China‐Australia Joint Research Centre for Infectious Diseases, School of Public Health Xi'an Jiaotong University Health Science Centre Xi'an China; ^7^ Artificial Intelligence and Modelling in Epidemiology Program, Melbourne Sexual Health Centre, Alfred Health Melbourne Australia; ^8^ Central Clinical School, Faculty of Medicine Monash University Melbourne Australia; ^9^ Department of Epidemiology and Biostatistics, College of Public Health Zhengzhou University Zhengzhou China; ^10^ National & Local Joint Engineering Research Center of Biodiagnosis and Biotherapy The Second Affiliated Hospital of Xi'an Jiaotong University Xi'an China; ^11^ Global Health Institute, School of Public Health Xi'an Jiaotong University Health Science Center Xi'an China; ^12^ Shaanxi Provincial Clinical Medical Research Center of Infectious Diseases Xi'an China; ^13^ Key Laboratory of Surgical Critical Care and Life Support (Xi'an Jiaotong University), Ministry of Education Xi'an China; ^14^ Karsh Division of Gastroenterology and Hepatology, Cedars‐Sinai Medical Center Los Angeles California USA

**Keywords:** COVID‐19, diabetic ketoacidosis, disparities, excess mortality, forecast model, hyperosmolar hyperglycemic state

## Abstract

**Background:**

During the pandemic, a notable increase in diabetic ketoacidosis (DKA) and hyperosmolar hyperglycemic state (HHS), conditions that warrant emergent management, was reported. We aimed to investigate the trend of DKA‐ and HHS‐related mortality and excess deaths during the pandemic.

**Methods:**

Annual age‐standardized mortality rates related to DKA and HHS between 2006 and 2021 were estimated using a nationwide database. Forecast analyses based on prepandemic data were conducted to predict the mortality rates during the pandemic. Excess mortality rates were calculated by comparing the observed versus predicted mortality rates. Subgroup analyses of demographic factors were performed.

**Results:**

There were 71 575 DKA‐related deaths and 8618 HHS‐related deaths documented during 2006–2021. DKA, which showed a steady increase before the pandemic, demonstrated a pronounced excess mortality during the pandemic (36.91% in 2020 and 46.58% in 2021) with an annual percentage change (APC) of 29.4% (95% CI: 16.0%–44.0%). Although HHS incurred a downward trend during 2006–2019, the excess deaths in 2020 (40.60%) and 2021 (56.64%) were profound. Pediatric decedents exhibited the highest excess mortality. More than half of the excess deaths due to DKA were coronavirus disease 2019 (COVID‐19) related (51.3% in 2020 and 63.4% in 2021), whereas only less than a quarter of excess deaths due to HHS were COVID‐19 related. A widened racial/ethnic disparity was observed, and females exhibited higher excess mortality than males.

**Conclusions:**

The DKA‐ and HHS‐related excess mortality during the pandemic and relevant disparities emphasize the urgent need for targeted strategies to mitigate the escalated risk in these populations during public health crises.

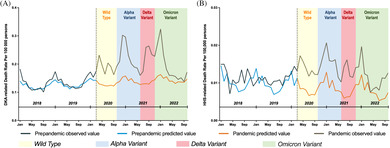

## Graphical abstract

Highlights

- The incidence and management of DKA and HHS, emergent complications of diabetes mellitus, were affected during the pandemic.
- In this study, we showed that DKA‐ and HHS‐related mortality increased dramatically during the pandemic, with widening age, sex, and racial/ethnic disparities.
- Our findings have implications for public health strategies to improve diabetes care and suggest a greater focus on these populations during future public health crises.

## 1 INTRODUCTION

The coronavirus disease 2019 (COVID‐19) pandemic has led to a surge in deaths globally, and the United States had the highest excess deaths at over one million deaths among 38 high‐income countries.^1^, ^2^ Importantly, it was reported that 25%–30% of these excess deaths were attributable to non‐COVID‐19 causes, such as complications of comorbid conditions, suggesting that indirect impacts of the pandemic on the health system might be at play.^3^ The COVID‐19 pandemic profoundly changed certain health‐seeking behaviors, disrupted access to medical care, and overstretched medical resources.^4^, ^5^, ^6^, ^7^

Disruptions along multiple points of the healthcare system exacerbated the management of chronic diseases that often require highly coordinated care and patients with diabetes mellitus (DM) were one of the most significantly affected groups.^8^, ^9^, ^10^ Previous studies have reported worsening glycemic control and delays in diabetes management during the pandemic.^11^ This in turn increased the incidence and mortality of diabetic emergencies including diabetic ketoacidosis (DKA) and hyperosmolar hyperglycemic state (HHS) among both adult and pediatric patients.^12^, ^13^, ^14^, ^15^ A meta‐analysis showed that severe DKA risk among pediatric patients was 44%–76% higher during the pandemic.^16^ DKA and HHS are life‐threatening emergencies with high fatalities, which occur in patients with decompensated DM and require intensive care unit‐level care. Before the COVID‐19 pandemic, mortality from DKA ranged from 5% to 10% and that from HHS ranged up to 10 times higher at 10%–50%.^17^, ^18^ The influx of severe COVID‐19 cases into the critical care units during the COVID‐19 pandemic largely limited their capacity, which further impacted the care of DKA and HHS. However, literature on mortality of DKA and/or HHS during the COVID‐19 pandemic is still limited.^13^, ^19^

Mortality is an important metric of the quality of care, and it can reflect the disparities in care among different patient demographics. Therefore, it is pivotal to recognize the extent of the pandemic's impact on DKA‐ and HHS‐related excess mortality. Our study aims to elucidate the trend of DKA‐ and HHS‐related mortality before and during the pandemic and to quantify the excess deaths during the pandemic. We also explored the disparities across different age, sex, and racial/ethnic groups.

## 2 RESEARCH DESIGN AND METHODS

### 2.1 Study design and study population

We conducted a cross‐sectional study based on the National Vital Statistics System dataset through the Vital Statistics Online Data Portal of the Centers for Disease Control (CDC) from January 1, 2006 to December 31, 2021.^20^ The database captures death records of over 99% of residents across the 50 United States and District of Columbia annually. The study obtained demographic data including age, sex, race/ethnicity, clinical status, and cause of death for each resident. As all data used in our study were both publicly available and deidentified, institutional review board approval for the study was not requested. The study is subject to the Strengthening the Reporting of Observational Studies in Epidemiology guidelines.

### 2.2 Definitions

We used *International Classification of Diseases, Tenth Revision* (ICD‐10) codes to define the cause of death listed in this study, including DKA (E10.1, E11.1, E12.1, E13.1, and E14.1), HHS (E10.0, E11.0, E12.0, E13.0, and E14.0), and U07.1 for COVID‐19. Decedents across all age groups were considered from January 1, 2006 to December 31, 2021. Each decedent's death certificate contains up to 20 related causes of death; the first position denotes the disease directly leading to death called “Underlying cause‐of‐death”, and the remaining causes of death are classified as “Multiple cause‐of‐death.” Because DKA and HHS are considered complications of DM, it may not always be identified as the underlying cause‐of‐death on the death certificate. To avert underestimation of death numbers, we utilized the multiple cause‐of‐death to characterize DKA‐ and HHS‐related mortality. To distinguish the multiple cause‐of‐death associated with DKA and HHS, these deaths were denoted as “diabetic ketoacidosis (DKA)‐related mortality” and “hyperosmolar hyperglycemic state (HHS)‐related mortality”, respectively. In addition, we also defined COVID‐19 as multiple cause‐of‐death. The analysis for race/ethnicity subgroup was up until the end of 2020 due to a modification in the race/ethnicity categories by CDC in 2021. The addition of a new category for individuals identifying as “more than one race” made the data for 2021 incompatible with that from 2020 and previous years.

### 2.3 Statistical analysis

Demographic characteristics of decedents with DKA and HHS were reported as frequencies with percentages. First, we calculated the DKA and HHS crude mortality rate by dividing the number of DKA‐ and HHS‐related deaths per year by the total US population in the corresponding year. Second, we computed the key outcome indicator as age‐standardized mortality rate (ASMR, per 100 000 persons), using direct method and a population from the 2010 US Census Standard Population as a reference. Our study divided age subgroups into four categories: 0–18 years, 19–44 years, 45–64 years, and ≥65 years. The first subgroup was stratified by single year of age, whereas the following three subgroups were stratified by both single year and five years of age from 19 to 85+. Excess deaths were estimated by comparing observed versus expected age‐standardized mortality rates ([observed – predicted]/predicted value × 100%) derived from mortality during 2006–2019 with linear and polynomial regression models. We tested time series regression models including autoregressive integrated moving average (ARIMA), autoregressive moving average model (ARMA), linear regression, and polynomial regression based on the ASMR observed from 2006 to 2019. The fitness of the regression models was decided by the RMSE (root mean square error) and *R* ^2^ (Table S1–S4). Mortality trends were examined by joinpoint piecewise regression with the use of annual percentage change (APC) to quantify the magnitude of the overall trend. This analysis was used to determine whether the trend was better explained by two segments or more and involved a piecewise linear regression using the grid search method, and the significance of the trend was determined through a Monte Carlo Permutation test (K scale from 0 to 4).^21^ Subgroup analyses by age, sex, race/ethnicity, and month were conducted. Race/ethnicity‐specific mortality rates were examined within the age groups. We excluded subgroups with less than 20 deaths to prevent inaccurate data and the possibility of reidentification.

All analyses were performed using National Cancer Institute's joinpoint regression (Joinpoint Trend Analysis Software version 4.9.1.0; National Cancer Institute, Bethesda, MD), R 4.0.2 statistical software (data management), and PyCharm 3.9.0 (predictive analysis). Two‐sided *p* value with a threshold of significance at 0.05 was used.

## 3 RESULTS

### 3.1 Decedent population and characteristics

A total of 71 575 DKA‐related deaths and 8618 HHS‐related deaths were documented from 2006 to 2021. The highest proportion of HHS‐related deaths occurred in the elderly (aged 65 or older) (61%), whereas the middle age group (aged 45–64) and the elderly contributed similarly to DKA‐related deaths. Males (56%) constituted a higher percentage of DKA‐related deaths than females (44%), but both sexes were comparable in terms of HHS‐related deaths. Non‐Hispanic Whites represented the majority of deaths (DKA, HHS: 63%, 65%), followed by non‐Hispanic Blacks and Hispanics (Tables S5 and S6).

### 3.2 Overall trend of excess deaths

**FIGURE 1:**
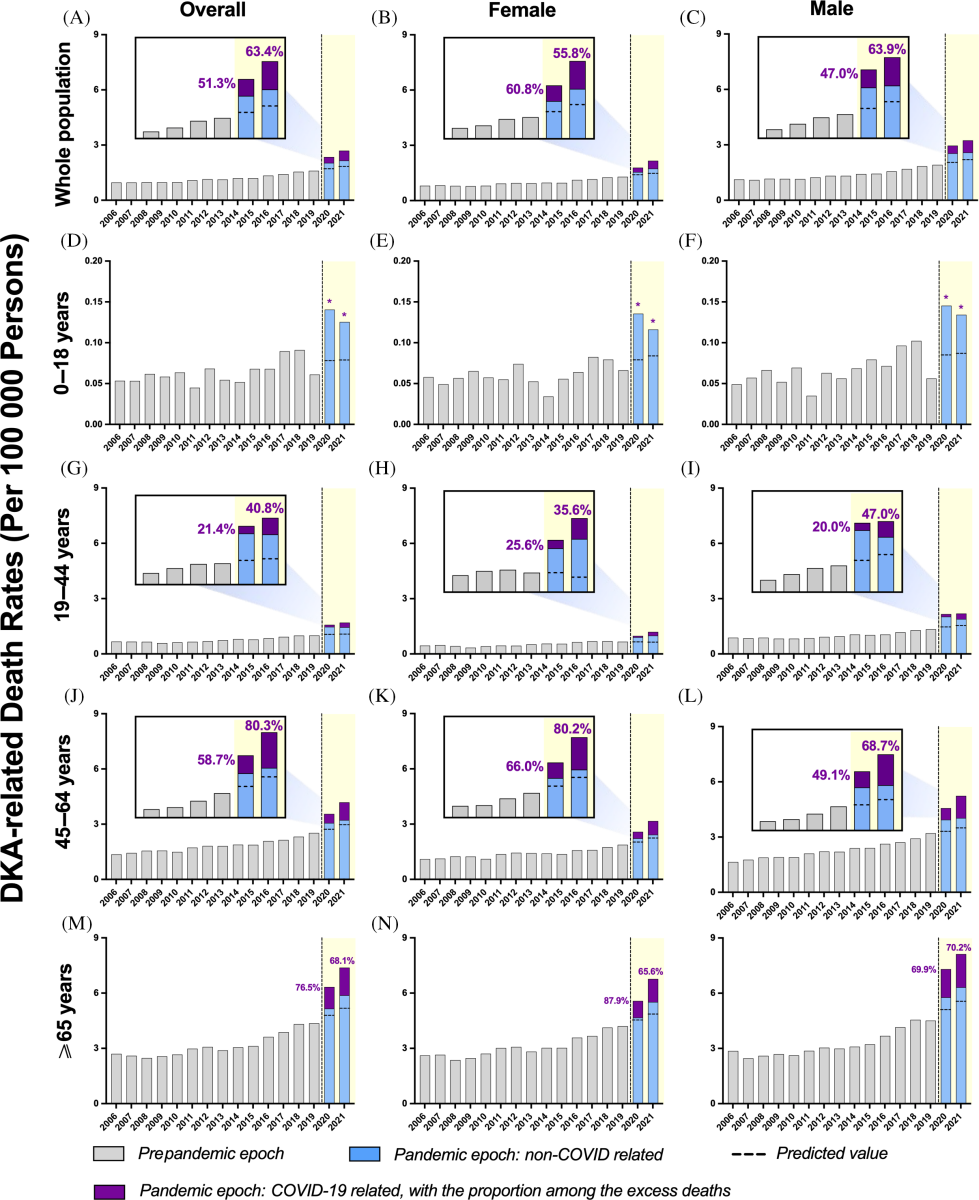
Temporal trends in diabetic ketoacidosis (DKA)‐related deaths and excess deaths during the coronavirus disease 2019 (COVID‐19) pandemic, by sex, further stratified by age. (A) Overall population, (B) female, (C) male, (D) aged 0–18 years in overall population, (E) aged 0–18 years in females, (F) aged 0–18 years in males, (G) aged 19–44 years in overall population, (H) aged 19–44 years in females, (I) aged 19–44 years in males, (J) aged 45–64 years in overall population, (K) aged 45–64 years in females, (L) aged 45–64 years in males, (M) aged 65 years and above in overall population, (N) aged 65 years and above in females, (O) aged 65 years and above in males. The observed mortality rates were above the predicted mortality rates (dashed horizontal line). DKA‐related death refers to multiple cause‐of‐death associated with DKA. *COVID‐19‐related death count less than nine persons.

**FIGURE 2:**
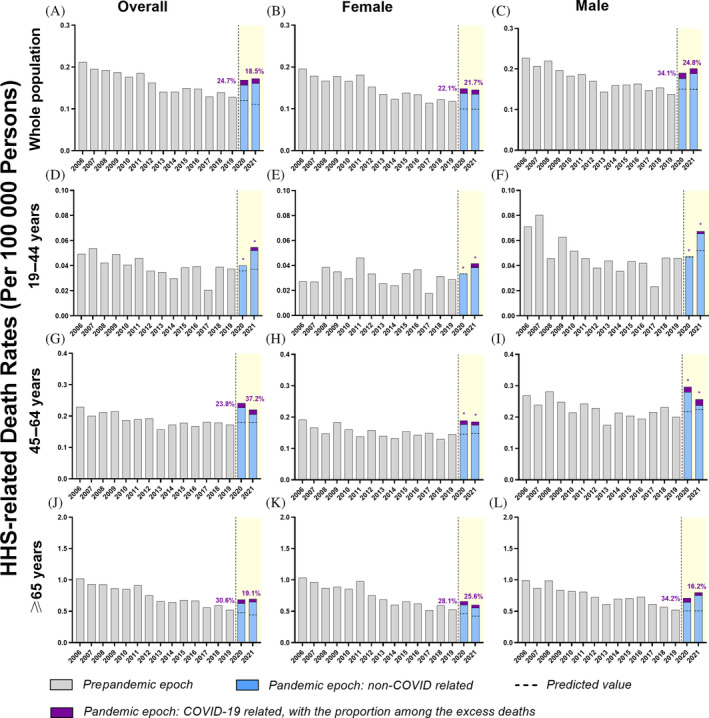
Temporal trends of hyperosmolar hyperglycemic state (HHS)‐related mortality and excess mortality during the coronavirus disease 2019 (COVID‐19) pandemic, by sex, further stratified by age. (A) Overall population, (B) female, (C) male, (D) aged 19–44 years in overall population, (E) aged 19–44 years in females, (F) aged 19–44 years in males, (G) aged 45–64 years in overall population, (H) aged 45–64 years in females, (I) aged 45–64 years in males, (J) aged 65 years and above in overall population, (K) aged 65 years and above in females, (L) aged 65 years and above in males. HHS‐related mortality rates (per 100 000 persons) increased across all subgroups during the pandemic. The observed mortality rates were above the predicted mortality rates (dashed horizontal line). HHS‐related death refers to multiple cause‐of‐death associated with HHS. *COVID‐19‐related death count less than nine persons.

Before the pandemic, a steady increase in deaths was observed for DKA, whereas HHS‐related deaths decreased steadily from 0.21 (per 100 000 persons) in 2006 to 0.13 in 2019 (Figures 1A and 2A). During the pandemic, there was a dramatic surge in DKA‐related deaths in 2020 (2.35; 36.91% excess mortality) that became even more prominent in 2021 (2.70; 46.58% excess mortality) (Table 1). On joinpoint analysis, the soar for 2019–2021 translated to an APC of 29.4% (95% CI: 16.0%–44.4%), which was noticeably higher than the modest increase between 2015 and 2019 (APC 8.6%; 95% CI: 2.8%–14.7%) (Table 1). More than half of the excess deaths were related to COVID‐19, with 51.3% for 2020 and 63.4% for 2021 (Figure 1A).

**TABLE 1:** Multiple cause‐of‐death and annual percentage change (APC) for diabetic ketoacidosis (DKA)‐related mortality in US adults by age group and sex, 2006–2021.

Age‐standardized mortality rate (per 100 000 persons)								Trend segment	
	2006 (prepandemic referent epoch)	2020 (Pandemic epoch 1)			2021 (Pandemic epoch 2)			Year	APC [95% CI]
		Observed	Predicted [95% CI]	% Increase^a^	Observed	Predicted [95% CI]	% Increase^a^		
Multiple cause‐of‐death^b^	0.96	2.35	1.72 [1.62 to 1.82]	**36.91**	2.70	1.84 [1.72 to 1.95]	**46.58**	2006–2015 2015–2019 2019–2021	3.0* [2.0 to 4.0] 8.6* [2.8 to 14.7] 29.4* [16.0 to 44.4]
Age									
0–18 years	0.05	0.14	0.08 [0.05 to 0.11]	**80.28**	0.13	0.08 [0.05 to 0.11]	**58.76**	2006–2014 2014–2019 2019–2021	0.8 [−3.7 to 5.5] 7.9 [−5.5 to 23.3] 28.0 [−15.9 to 94.8]
19–44 years	0.67	1.58	1.05 [0.95 to 1.15]	**50.30**	1.70	1.08 [0.94 to 1.22]	**57.45**	2006–2009 2009–2019 2019–2021	−5.1** [−8.6 to −1.3] 5.8* [5.1 to 6.6] 32.4* [22.6 to 43.1]
45–64 years	1.36	3.56	2.72 [2.48 to 2.96]	**30.79**	4.19	2.98 [2.63 to 3.32]	**40.63**	2006–2016 2016–2019 2019–2021	3.8* [2.8 to 4.9] 8.1 [−4.7 to 22.7] 30.1* [14.7 to 47.7]
≥65 years	2.70	6.34	4.79 [4.34 to 5.24]	**32.29**	7.38	5.17 [4.65 to 5.69]	**42.75**	2006–2014 2014–2019 2019–2021	2.2** [0.4 to 4.1] 8.7* [3.1 to 14.6] 29.1* [9.2 to 52.5]
Sex									
Female	0.80	1.79	1.39 [1.27 to 1.52]	**28.73**	2.27	1.49 [1.35 to 1.62]	**52.04**	2006–2009 2009–2019 2019–2021	−1.1 [−8.6 to 7.1] 5.0* [3.5 to 6.5] 34.0* [14.3 to 57.0]
Male	1.13	2.96	2.06 [1.97 to 2.16]	**43.53**	3.25	2.21 [2.10 to 2.32]	**46.90**	2006–2015 2015–2019 2019–2021	3.1* [2.2 to 4.1] 9.2* [3.8 to 14.9] 30.4* [17.8 to 44.3]

*Note*: Bold indicates the proportion values for the increase in mortality, and the formula is as follows: Increase%=(observed value ‐ predicted value)/predicted value*100%.

Abbreviations: CI: Confidence Interval.

^a^ % of increase from predicted to observed value.

^b^ Multiple cause‐of‐death: deaths caused by various causes including DKA.

* *p* value <0.01;

** *p* value <0.05.

Table 2 showed a similar rise in HHS‐related mortality during the pandemic, which was identical for 2020 and 2021 (0.169, 0.172) (Figure 2A). However, due to the decreasing trend in mortality before the pandemic, the HHS‐related excess deaths rose from 40.60% in 2020 to 56.64% in 2021. Overall, in contrast to DKA‐related deaths, only less than a quarter of HHS‐related excess deaths during the pandemic were related to COVID‐19 (24.7% for 2020, 18.5% for 2021) (Figure 2A). Of note, more than 70% of DKA‐related excess deaths among the elderly decedents were associated with COVID‐19 (Figure 1M), whereas only 20%–40% of DKA‐related excess deaths were associated with COVID‐19 for young adults (Figure 1G). There was a rise in the percentage of COVID‐19‐related deaths in middle‐aged adults, which rose from 58.7% in 2020 to 80.3% in 2021 (Figure 1J). However, the COVID‐19‐associated mortality rates for pediatrics were not estimated, because of the small sample size (Figure 1D–F).

**TABLE 2:** Multiple cause‐of‐death and annual percentage change (APC) for hyperosmolar hyperglycemic state (HHS)‐related mortality in US adults by sex and age group, 2006–2021.

Age‐standardized mortality rate (per 100 000 persons)								Trend segment	
	2006 (prepandemic referent epoch)	2020 (Pandemic epoch 1)			2021 (Pandemic epoch 2)			Year	APC [95% CI]
		Observed	Predicted [95% CI]	% Increase^a^	Observed	Predicted [95% CI]	% Increase^a^		
Multiple cause‐of‐death^b^	0.21	0.168	0.12 [0.09 to 0.14]	**40.60**	0.172	0.11 [0.09 to 0.13]	**56.64**	2006–2019 2019–2021	−3.6* [−4.5 to −2.7] 19.4 [−0.3 to 43.0]
Age									
0–18 years	NA	NA	NA	NA	NA	NA	NA	NA	NA
19–44 years	0.05	0.04	0.04 [0.02 to 0.05]	**11.47**	0.05	0.04 [0.02 to 0.06]	**47.55**	2006–2017 2017–2021	−5.3* [−7.7 to −2.8] 15.4** [2.3 to 30.3]
45–64 years	0.23	0.24	0.18 [0.15 to 0.21]	**33.95**	0.22	0.18 [0.15 to 0.22]	**22.38**	2006–2016 2016–2021	−3.0* [−4.9 to −1.0] 6.7** [0.7 to 13.0]
≥65 years	1.02	0.69	0.48 [0.36 to 0.59]	**42.83**	0.69	0.44 [0.32 to 0.56]	**57.80**	2006–2019 2019–2021	−4.7* [−5.7 to −3.7] 15.7 [−4.8 to 40.6]
Sex									
Female	0.20	0.15	0.10 [0.08 to 0.13]	**47.80**	0.15	0.10 [0.07 to 0.13]	**46.66**	2006–2018 2018–2021	−4.2* [−5.5 to −2.8] 8.7 [−3.5 to 22.4]
Male	0.23	0.19	0.15 [0.11 to 0.18]	**26.52**	0.20	0.15 [0.11 to 0.18]	**34.08**	2006–2019 2019–2021	−3.3* [−4.4 to −2.2] 23.0 [−0.5 to 51.9]

*Note*: Bold indicates the proportion values for the increase in mortality, and the formula is as follows: Increase%=(observed value ‐ predicted value)/predicted value*100%.

Abbreviations: CI: Confidence Interval; NA: data not available.

^a^ % of increase from predicted to observed value.

^b^ Multiple cause‐of‐death: deaths caused by various causes including HHS.

* *p* value <0.01;

** *p* value <0.05.

### 3.3 Mortality by age and sex during the pandemic

For DKA‐related deaths, although elderly decedents (age 65 and older) had the highest ASMR during the pandemic (Figure 1M–O), pediatric decedents exhibited the highest percentage difference between observed and predicted mortality rates (80.28% for 2020 and 58.76% for 2021), followed by young adults (50.30% for 2020 and 57.45% for 2021) (Table 1). For both male and female pediatric decedents, the excess deaths were more prominent in 2020 compared with 2021, with both dropping from more than 70% to below 50% (Table 3). Although male decedents bore a higher ASMR than their female counterparts across all age subgroups (Figure 1B,C,E,F,H,I,K,L,N,O), overall, females exhibited a higher relative excess mortality than males during the pandemic (Table 1). In addition, there was a delayed rise in mortality for female decedents from 28.73% in 2020 to 52.04% in 2021. A similar delay was also observed in young female adults (Tables 1 and 3).

**TABLE 3:** Multiple cause‐of‐death and annual percentage change (APC) for diabetic ketoacidosis (DKA)‐related and hyperosmolar hyperglycemic state (HHS)‐related mortality in US adults by sex and age group, 2006–2021.

Age‐standardized mortality rate (per 100 000 persons)								Trend segment	
	2006 (prepandemic referent epoch)	2020 (Pandemic epoch 1)			2021 (Pandemic epoch 2)			Year	APC [95% CI]
		Observed	Predicted [95% CI]	% Increase^a^	Observed	Predicted [95% CI]	% Increase^a^		
DKA									
Female									
0–18 years	0.06	0.14	0.08 [0.04 to 0.12]	**71.73**	0.12	0.08 [0.04 to 0.13]	**38.31**	2006–2021	4.4* [1.2 to 7.7]
19–44 years	0.46	0.98	0.67 [0.54 to 0.80]	**46.58**	1.19	0.63 [0.44 to 0.81]	**89.39**	2006–2009 2009–2019 2019–2021	−7.4 [−14.3 to 0.1] 6.8** [5.3 to 8.4] 27.8** [9.4 to 49.2]
45–64 years	1.10	2.59	2.04 [1.73 to 2.34]	**26.82**	3.17	2.24 [1.80 to 2.68]	**41.38**	2006–2015 2015–2019 2019–2021	3.0** [1.1 to 4.9] 6.7 [−3.4 to 17.8] 31.1* [7.5 to 59.9]
≥65 years	2.61	5.58	4.54 [4.01 to 5.07]	**22.84**	6.77	4.86 [4.25 to 5.48]	**39.26**	2006–2015 2015–2019 2019–2021	2.6* [0.7 to 4.6] 8.8 [−2.0 to 20.8] 25.0* [1.4 to 54.1]
Male									
0–18 years	0.05	0.15	0.09 [0.05 to 0.12]	**70.99**	0.13	0.09 [0.05 to 0.13]	**54.35**	2006–2021	5.8** [2.6 to 9.1]
19–44 years	0.88	2.17	1.44 [1.35 to 1.54]	**50.45**	2.20	1.55 [1.44 to 1.66]	**41.76**	2006–2017 2017–2021	2.6** [0.9 to 4.3] 20.5** [11.5 to 30.2]
45–64 years	1.65	4.57	3.29 [3.09 to 3.48]	**38.97**	5.24	3.48 [3.25 to 3.70]	**50.46**	2006–2016 2016–2019 2019–2021	4.3** [3.6 to 5.0] 8.4 [−0.3 to 17.8] 29.5** [19.2 to 40.7]
≥65 years	2.86	7.31	5.11 [4.63 to 5.59]	**42.99**	8.12	5.55 [5.00 to 6.10]	**46.38**	2006–2014 2014–2019 2019–2021	2.0* [0.1 to 4.0] 10.2** [4.2 to 16.4] 32.4** [11.1 to 57.8]
HHS									
Female									
0–18 years	NA	NA	NA	NA	NA	NA	NA	NA	NA
19–44 years	0.03	0.03	0.03 [0.01 to 0.06]	**−0.45**	0.04	0.04 [0.00 to 0.75]	**7.12**	2006–2019 2019–2021	−1.3 [−5.2 to 2.7] 22.0 [−43.3 to 162.8]
45–64 years	0.19	0.19	0.15 [0.12 to 0.17]	**30.19**	0.19	0.15 [0.11 to 0.18]	**24.88**	2006–2018 2018–2021	−2.1* [−3.8 to −0.4] 13.3 [−1.9 to 30.9]
≥65 years	1.03	0.66	0.46 [0.38 to 0.54]	**43.80**	0.60	0.42 [0.33 to 0.51]	**44.35**	2006–2021	−4.1** [−5.4 to −2.9]
Male									
0–18 years	NA	NA	NA	NA	NA	NA	NA	NA	NA
19–44 years	0.07	0.05	0.05 [0.03 to 0.07]	**−1.09**	0.07	0.05 [0.03 to 0.08]	**29.97**	2006–2017 2017–2021	−6.9** [−10.0 to −3.7] 19.5* [2.0 to 40.0]
45–64 years	0.27	0.30	0.22 [0.17 to 0.26]	**36.60**	0.26	0.22 [0.17 to 0.28]	**15.18**	2006–2013 2013–2019 2019–2021	−4.5* [−7.5 to −1.3] 1.7 [−3.7 to 7.4] 12.5 [−11.7 to 43.3]
≥65 years	0.99	0.71	0.51 [0.43 to 0.58]	**40.12**	0.80	0.51 [0.43 to 0.58]	**57.41**	2006–2019 2019–2021	−4.2** [−5.4 to −2.9] 21.1 [−5.5 to 55.3]

*Note*: Bold indicates the proportion values for the increase in mortality, and the formula is as follows: Increase%=(observed value ‐ predicted value)/predicted value*100%.

Abbreviations: CI: Confidence Interval; NA: data not available.

^a^ % of increase from predicted to observed value.

* *p* value<0.05;

** *p* value<0.01.

For HHS‐related deaths, elder decedents (those aged 65 and above) had the highest ASMR and the highest percentage difference between observed and expected mortality (42.83% for 2020 and 57.80% for 2021) (Figure 2J and Table 2). Male and female elderly decedents contributed similarly to this prominent rise (Figure 2K,L and Table 3). In contrast, there were too few deaths for pediatric decedents for analysis. Notably, young adults experienced a delayed surge in mortality for both males and females (Figure 2D,E,F and Table 3). Females exhibited a higher excess mortality than males (47.80% vs. 26.52% in 2020 and 46.66% vs. 34.08% in 2021, respectively) (Figure 2B,C and Table 2). Both female and male middle‐aged decedents demonstrated a positive shift in excess mortality in 2021 (Tables 2 and 3), with the excess deaths attributed to COVID‐19 being too small to estimate (Figure 2H,I). The middle‐aged decedents showed a 23.8% and 37.2% excess deaths related to COVID‐19 in the years 2020 and 2021, respectively (Figure 2G).

### 3.4 Mortality by ethnicity

For DKA, non‐Hispanic Blacks and non‐Hispanic American Indian/Alaska Natives (AI/AN) suffered the highest DKA‐related mortality among all racial/ethnic groups from 2006 to 2020, whereas non‐Hispanic Asians had the lowest mortality (Figure S1A). In 2020, all racial/ethnic groups saw a significant rise in DKA‐related mortality with a widening disparity. The most dramatic rise in relative mortality was observed in non‐Hispanic American Indian/Alaska Natives (151.2% excess mortality), Hispanics (77.0%) and non‐Hispanic Blacks (62.9%) (Table S7).

For HHS, non‐Hispanic Blacks also had the highest mortality compared with other racial/ethnic groups (Figure S1B). Excess deaths related to HHS were the most significant for Hispanics (133.1%), non‐Hispanic Asians (51.1%), non‐Hispanic Blacks (26.6%), and non‐Hispanic Whites (24.1%) (Table S8). Taken together, non‐Hispanic Whites had the lowest excess mortality in both DKA‐ (19.9%) and HHS‐related (24.1%) deaths. Different racial/ethnic‐age groups among DKA had similar trends in mortality among age subgroups (Table S9).

### 3.5 Surge of mortality by SARS‐CoV‐2 variant

**FIGURE 3:**
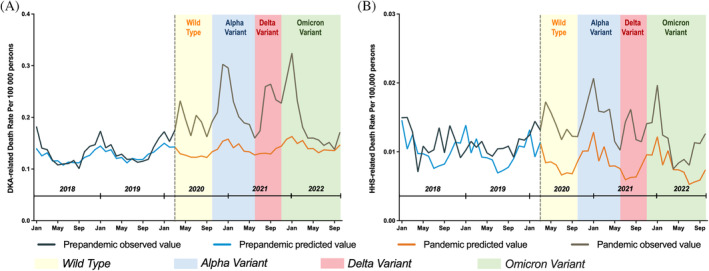
Monthly age‐standardized mortality for diabetic ketoacidosis (DKA) and hyperosmolar hyperglycemic state (HHS) in the United States in 2018–2021. (A) Overall mortality for decedents with DKA, (B) overall mortality for decedents with HHS.

As illustrated in Figure 3A, the surges of DKA‐related mortality coincided with the emergence of evolving variants of SARS‐CoV‐2, resulting in distinct wild‐type outbreak, Alpha variant outbreak, Delta variant outbreak, and Omicron variant outbreak. Noticeably, the Omicron outbreak plunged after a short‐lived peak and continued to drop until it reached the level of predicted mortality by July 2022. HHS‐related mortality also presented similar outbreaks corresponding to different variants (Figure 3B). There was a drop in mortality in the Omicron outbreak, followed by a slight rise as it reached July 2022.

## 4 DISCUSSION

In this first nationwide temporal trend study of DKA‐ and HHS‐related mortality in the United States to investigate the survival impact of the COVID‐19 pandemic, we showed a near 40% of excess deaths related to DKA and 50% of excess deaths related to HHS during the pandemic in the United States. The percentages were far higher than that of multiple causes of mortality (around 20%), signifying the tremendous impact the pandemic had on diabetic emergencies.^22^ Before the pandemic, DKA‐related mortality experienced a steady upward trend, whereas HHS‐related mortality witnessed a continuous decline and was much lower than that of DKA. Although the COVID‐19 pandemic contributed to the increase in mortality for both DKA and HHS, the majority of HHS‐related excess deaths that occurred during this time were not associated with COVID‐19, whereas more than half of the DKA‐related excess deaths overall were related to COVID‐19. The percentage was highest among elderly decedents (more than 70%) and declined with younger age, with only 20%–40% of excess deaths related to COVID‐19. Our study also highlighted a widening age and racial/ethnic disparity in mortality during the pandemic. Non‐Hispanic American Indian/Alaska Native and Hispanics suffered a surge in DKA‐related mortality around three times that of non‐Hispanic Whites. In terms of HHS‐related mortality, non‐Hispanic Blacks demonstrated a mortality rise nearly five times that of non‐Hispanic Whites. We also illustrated the outbreaks of mortality that corresponded to different SARS‐CoV‐2 variants and the significant drop in mortality in the postpandemic era. In essence, our study suggested that the COVID‐19 pandemic contributed to a significant increase in DKA‐ and HHS‐related mortality and further accentuated the sex and racial/ethnic disparities.

During the COVID‐19 pandemic, our study observed a surge in DKA‐related (nearly 40% excess mortality) and HHS‐related (50% excess mortality) mortality. Although mortality risk from HHS (10%–50%) has been reported to be 10 times that of DKA before the pandemic,^17^, ^18^, ^23^ we showed that even before the pandemic, the DKA‐related mortality was consistently higher than that of HHS. The same held true as the United States entered the pandemic era. This could potentially be explained by our study design. We applied a broader definition of DKA‐related and HHS‐related deaths, which is an umbrella term for all deaths that have DKA or HHS recorded as one of the causes on the death certificates. In contrast to previous studies, we took this approach to include deaths that were indirectly caused by DKA, HHS, and/or COVID‐19. There was also a steeper surge in mortality for DKA compared with HHS during the pandemic, as evidenced by its significant APC of 29.4% (16.0%–44.4%). This brought DKA‐related mortality to 2.35 per 100 000 persons in 2020 and 2.70 in 2021 and HHS‐related mortality to 0.17 in both 2020 and 2021. Our findings corroborate the study by Pasquel et al. using Glytec US national database including 17 states, which found that DKA patients with SARS‐CoV‐2 infection had significantly elevated in‐hospital mortality compared with those without COVID‐19 (19% vs. 2%).^13^ Similarly, a small case series led by Pal et al. also showed a noticeable mortality as high as 50% in DKA patients with COVID‐19.^19^

The bidirectional relationship between COVID‐19 and DM has been extensively studied since the beginning of the pandemic. COVID‐19 was found to unmask previously undiagnosed DM and increase the risk of new‐onset diabetes.^24^ It has also been associated with increased in‐hospital mortality, especially for those with type 2 DM or new‐onset DM.^10^, ^14^, ^19^ In particular, SARS‐CoV‐2 infection increases the risk and severity of DKA through various pathways.^24^, ^25^ However, literature that investigated the association of SARS‐CoV‐2 infection with HHS was scarce and limited to case‐series and case report design.^24^, ^26^ In addition, corticosteroids given as part of the treatment for SARS‐CoV‐2 infection may further aggravate the severity of DKA and HHS by exacerbating hyperglycemia. Lastly, it is important to take into consideration the changes in health behaviors during the pandemic. There was an increase in psychological stress, depression, weight gain, physical inactivity, medication noncompliance, and less healthy dietary habits when the pandemic hit, which greatly exacerbated metabolic diseases such as DM.^4^ During the pandemic, outpatient visits were delayed,^5^ and laboratory testing declined dramatically for DM and other chronic diseases,^6^, ^7^ which, along with the stay‐at‐home mandates, greatly disrupted the delivery of care. Intriguingly, although we showed that more than half of DKA‐related excess deaths were related to COVID‐19, our study found that more than three‐quarters of HHS‐related deaths were not attributable to COVID‐19 infection itself. This could be explained by the diversion of medical resources, especially intensive care, from chronic diseases to the rapidly escalating COVID‐19‐pandemic. The pandemic could also have resulted in the delayed presentation of DKA and HHS patients to the appropriate medical services, or even underdiagnosis, as patients were reluctant to seek help for their own medical conditions for the fear of contracting COVID‐19 in the hospital settings.^7^

The mortality rate for DKA and HHS among different age groups is also of great interest, as COVID‐19 mortality is strongly associated with age.^3^ Our study showed that the elderly had the highest mortality before and during the pandemic for both diseases, consistent with previous literature.^2^, ^13^, ^27^ However, we observed the most prominent surge in DKA‐related deaths among young adults and pediatric decedents. This is similar to a previous US report by Mulligan et al. that used the same CDC WONDER nationwide database from April 2020 to December 2021 to investigate multiple causes of mortality during the pandemic, which showed that young adults had a higher percentage of excess deaths than older adults.^22^ Breaking down DKA‐related excess deaths by age group, the majority of the elderly excess deaths were related to COVID‐19 (more than 70%), and most of the young decedent excess deaths were not associated with COVID‐19 (60%–80%). This is consistent with Mulligan et al.'s study, which showed that most COVID‐19 excess deaths occur in the elderly, whereas young adults were mostly affected by non‐COVID‐19 deaths. As is also shown in a previous report, among patients without SARS‐CoV‐2 infection, more than 60% of DKA cases occur in young adults.^28^ This could also be explained that DKA is more associated with type 1 DM, which presents at an earlier age, often in adolescence. For pediatric patients, studies have shown an increase in DKA and severe DKA risks among children with type 1 DM, but there exist no studies so far that explore pediatric mortality trends.^16^

On the other hand, there was a more delayed but most prominent surge in HHS‐related mortality among young adults, which may suggest the effect of pandemic‐associated disruption of routine diabetes care. Studies have shown that a large number of young adults skipped or delayed medical care during the pandemic, and some of them do not have insurance coverage.^4^, ^29^ They had poorer adherence to insulin protocols^30^ and COVID‐19 prevention measures and were less likely to meet glycemic control targets than older patients.

In terms of the difference in mortality by sex, our results are in line with Desai et al.'s 10‐year longitudinal study based on a US nationwide database, which showed a higher incidence, admission, and mortality of DKA in males than females.^30^ A cross‐sectional registry‐based study encompassing 7012 adult patients with type 1 DM from 70 US practices in 2010 reported that females experienced a higher prevalence of DKA compared with males,^31^ whereas a systematic review of 24 000 children from 31 countries revealed no effect of sex on the frequency of DKA.^32^ When compared with males, females are more likely to comply with restraining public health measures and less likely to pursue smoking and alcohol, and more females recognize the pandemic as a more serious problem.^33^ Furthermore, a recent meta‐analysis reported that SARS‐CoV‐2‐infected males have nearly three times the odds of requiring intensive care and 1.39 the odds of mortality compared with SARS‐CoV‐2‐infected females.^34^ Interestingly, despite these phenomena, females have actually been shown to achieve poorer glycemic control than males in multiple studies.^35^ Our study showed that during the pandemic, females actually exhibited a higher relative excess mortality than their male counterparts for both DKA‐ and HHS‐related deaths. This implies an augmented sex disparity during the pandemic. More studies are warranted to explain the widening disparity.

In regards to ethnicity, we showed that the racial/ethnic disparity was accentuated during the pandemic, with a distressing upward trend of mortality rates in non‐Hispanic Blacks, Hispanics, and AI/AN who already had the highest mortality even before the COVID‐19 pandemic. On the contrary, although non‐Hispanic Whites constituted more than 60% of decedents for both DKA‐ and HHS‐related deaths, they had the lowest increase in mortality rates related to these two conditions. Although non‐Hispanic Blacks and Asians develop DM at a younger age and have a higher prevalence of type 2 DM, non‐Hispanic Asians consistently had the lowest DKA‐ and HHS‐related mortality throughout the study period.^36^ Our findings complement recent literature showing that non‐Hispanic Blacks had the highest prevalence of COVID‐19 infection and ketosis‐prone DM^36^ and constituted the highest proportion of COVID‐19 patients with DKA during the pandemic.^19^ The socioeconomic and health inequity experienced by non‐Hispanic Blacks and Hispanics was disproportionately widened during the pandemic.^37^ Not only did they have a higher prevalence of diabetes and obesity but they also sustained an even higher occupational risk and significant loss of healthcare coverage, which exacerbated poorer glycemic control and health maintenance.^38^

To our knowledge, this is the first nationwide temporal study on hyperglycemic emergency mortality in the United States that spans over 16 years. To present the long‐term longitudinal mortality analysis, we used a CDC nationwide database that contains more than 99% of deaths in 51 US states. Our study also benefited from predictive analyses and quantification of mortality trends during the pandemic. Another unique part of our study is the presentation of causes of death and subgroup analyses by age, sex, and racial/ethnic groups, which allows for the interpretation of the direct and indirect effect of the pandemic on mortality. There are a few limitations to consider, however. First, underreporting of DKA and HHS and underdiagnosis of new‐onset DM during the chaos of the pandemic is possible, and the presence of acute kidney injury may lead to overdiagnosis of DKA due to subsequent high anion‐gap metabolic acidosis. This could lead to misclassification of cause of death on the death certificates. Second, combined DKA/HHS and euglycemic DKA (EDKA) are distinct disease entities within DKA, with DKA/HHS making up 20%–30% of DKA.^39^ DKA/HHS presents with a higher mortality than pure DKA.^39^ Our study is based on national registry data utilizing ICD‐10 coding, in which DKA/HHS and EDKA fall under the category of DKA. Thus, overestimation of DKA mortality is a possibility. Third, the COVID‐19 pandemic started in the United States in March 2020. Therefore, the year 2020 includes 3 months prepandemic and could lead to underestimation of mortality data for 2020. Lastly, the WONDER database did not contain information such as the duration of DM or use of corticosteroids. This leaves room for future investigation.

## 5 CONCLUSION

In conclusion, our study showed that 40% of excess deaths were related to DKA and 50% of excess deaths were related to HHS during the COVID‐19 pandemic in the United States. DKA and HHS are not only important complications of DM but they could also serve as one of the quality indicators in intensive care medicine during the pandemic. The astonishing surge in DKA‐related mortality and the exaggerated sex/racial/ethnic disparities call for particular attention. Our findings have important implications for public health strategies to reinforce healthcare improvement initiatives and diabetes care surveillance during a national health crisis. Because the population susceptible to DKA and HHS are different, it is important to heighten the care for type 1 DM, ketosis‐prone DM, and to recognize that type 2 DM can also develop DKA. Particular attention should be drawn to maintaining the quality of diabetes care for children and young adults, which presented the highest relative excess mortality. Medical resources, such as manpower and equipment, have been diverted due to the increased number of patients with COVID‐19. This overstretch of resources could lead to dangerous lapses in standards of care for patients with DKA and HHS. Our study may serve as a reminder that proper allocation of medical resources and tightened infection control are crucial in preparation for future public health crises.

## AUTHOR CONTRIBUTIONS

All authors contributed to data interpretation and approved the manuscript. XH, AHH, FL, XG, JZ, LZ, FJ, and YHY designed the study. JZ, FJ, and YHY designed the methodology. XH, FL, XG, YG, YL, XH, and JX analyzed the data. XH, AHH, and YHY drafted the manuscript. NG, YJ, YW, JZ, LZ, FJ, and YHY contributed to the critical review of the manuscript. FJ and YHY contributed to the critical revision of the manuscript. FJ and YHY contributed to study conception and study supervision.

## FUNDING INFORMATION

No external funding to disclose.

## CONFLICT OF INTEREST STATEMENT

Fanpu Ji receives speaker fees from Gilead Sciences, MSD and Ascletis and is a consultant/advisory board member for Gilead, MSD. All other authors do not have any conflict of interest.

## Supporting information


**Data S1.** Supporting information.

## Data Availability

The datasets generated during and/or analyzed in the current study are available in the Statistics NCfH. Vital Statistics Online Data Portal repository, https://www.cdc.gov/nchs/data_access/vitalstatsonline.htm#Mortality_Multiple.
