# Supplementary material for: Trends in diabetic ketoacidosis‐ and hyperosmolar hyperglycemic state‐related mortality during the COVID‐19 pandemic in the United States: A population‐based study
Source: J Diabetes. 2024 Aug 13;16(8):e13591. doi: 10.1111/1753-0407.13591 (PMC11320749; doi:10.1111/1753-0407.13591)

**Online-Only Supplementary Material**

**Trends in Diabetic Ketoacidosis and Hyperosmolar Hyperglycemic State-related Mortality during the COVID-19 Pandemic in the United States: A population-based study**

Xinyuan He^#^, Amy Huaishiuan Huang^#^, Fan Lv^#^, Xu Gao, Yuxin Guo, Yishan Liu, Xiaoqin Hu, Jingyi Xie, Ning Gao, Yang Jiao, Yuan Wang, Jian Zu, Lei Zhang, Fanpu Ji^*^, Yee Hui Yeo^*^

**Table of Content**

**Supplementary Table 1.** Different model RMSE in DKA-related mortality in U.S. adults between 2006-2019.

**Supplementary Table 2.** Different model RMSE in HHS-related mortality in U.S. adults between 2006-2019.

**Supplementary Table 3.** Model parameter in DKA-related mortality in U.S. adults between 2006-2019.

**Supplementary Table 4.** Model parameter in HHS-related mortality in U.S. adults between 2006-2019.

**Supplementary Table 5.** Characteristics of DKA-related death in the United States, 2006-2021.

**Supplementary Table 6.** Characteristics of HHS-related death in the United States, 2006-2021.

**Supplementary Table 7**. Age-standardized mortality rate and annual percentage change (APC) in DKA-related mortality among U.S. adults, between 2006 and 2020, by race/ethnicity.

**Supplementary Table 8.** Age-standardized mortality rate and annual percentage change (APC) in HHS-related mortality among U.S. adults, between 2006 and 2020, by race/ethnicity.

**Supplementary Table 9.** Age-standardized mortality rate and annual percentage change (APC) in DKA-related mortality among U.S. adults, between 2006 and 2020, by race/ethnicity and age.

**Supplementary Fig.1.** Observed age-standardized mortality rates for DKA and HHS-related deaths before and during the COVID-19 pandemic by race/ethnicity.

**Supplementary Table 1. Different model RMSE in DKA-related mortality in U.S. adults between 2006-2019.**

|  | **ARMA (ARIMA)**  **(RMSE)** | **OLS**  **(RMSE)** |
| --- | --- | --- |
| **Overall** | | |
| Total | ARIMA(0,2,1)  (0.050) | $y=0.973-0.013x+0.004x^{2}$  (0.029) |
| **Age** | | |
| 0-18 years | ARIMA(0,0,0)  (0.012) | $y=0.06-0.002x+0.0002x^{2}$  (0.010) |
| 19-44 years | ARIMA(0,1,0)  (0.039) | $y=0.74-0.068x+0.011x^{2}-$0.0003$x^{3}$  (0.021) |
| 45-64 years | ARIMA(0,1,0)  (0.090) | $y=1.26+0.11x-0.01x^{2}+$0.0006$x^{3}$  (0.052) |
| ≥65 years | ARIMA(0,1,0)  (0.192) | $y=2.72-0.08x+0.015x^{2}$  (0.133) |
| **Sex** | | |
| Female | ARIMA(0,1,0)  (0.053) | $y=0.81-0.009x+0.003x^{2}$  (0.035) |
| Male | ARIMA(0,2,1)  (0.055) | $y=0.24-0.014x+0.0005x^{2}$  (0.028) |
| **Race and ethnicity** | | |
| Non-Hispanic Whites | ARIMA(0,2,1)  (0.041) | $y=0.82-0.002x+0.004x^{2}$  (0.026) |
| Non-Hispanic Blacks | ARIMA(0,1,0)  (0.210) | $y=2.29-0.08x+0.008x^{2}$  (0.115) |
| Hispanics | ARIMA(0,1,0)  (0.107) | $y=1.02-0.0007x+0.003x^{2}$  (0.069) |
| Non-Hispanic AI/AN | ARIMA(0,1,0)  (0.369) | $y=2.04-0.30x+0.058x^{2}-$0.003$x^{3}$  (0.208) |
| Non-Hispanic Asians | ARIMA(0,1,0)  (0.081) | $y=0.61-0.047x+0.0048x^{2}$  (0.046) |
| **Female by age** | | |
| 0-18 years | ARIMA(0,0,0)  (0.012) | $y=0.06-0.002x+0.0002x^{2}$  (0.010) |
| 19-44 years | ARIMA(0,1,0)  (0.051) | $y=0.57-0.10x+0.016x^{2}-$0.0006$x^{3}$  (0.028) |
| 45-64 years | ARIMA(0,1,0)  (0.102) | $y=0.99+0.10x-0.011x^{2}+$0.0006$x^{3}$  (0.066) |
| ≥65 years | ARIMA(0,1,0)  (0.222) | $y=2.64-0.06x+0.012x^{2}$  (0.157) |
| **Male by age** | | |
| 0-18 years | ARIMA(0,1,0)  (0.020) | $y=0.047+0.0025x$  (0.014) |
| 19-44 years | ARIMA(0,2,1)  (0.051) | $y=0.90-0.030x+0.004x^{2}$  (0.029) |
| 45-64 years | ARIMA(0,1,0)  (0.089) | $y=1.68+0.03x+0.005x^{2}$  (0.058) |
| ≥65 years | ARIMA(0,1,0)  (0.225) | $y=2.83-0.12x+0.018x^{2}$  (0.142) |

**Supplementary Table 2. Different model RMSE in HHS-related mortality in U.S. adults between 2006-2019.**

|  | **ARMA (ARIMA)**  **(RMSE)** | **OLS**  **(RMSE)** |
| --- | --- | --- |
| **Overall** | | |
| Total | ARIMA (0,1,0)  (0.014) | $y=0.21-0.006x$  (0.0098) |
| **Age** | | |
| 19-44 years | ARIMA (0,1,0)  (0.010) | $y=0.055-0.003x+0.0002x^{2}$  (0.0065) |
| 45-64 years | ARIMA (0,1,0)  (0.014) | $y=0.23-0.01x+0.0005x^{2}$  (0.0085) |
| ≥65 years | ARIMA (0,1,0)  (0.059) | $y=1.03-0.04x$  (0.043) |
| **Sex** | | |
| Female | ARIMA (0,1,0)  (0.015) | $y=0.20-0.006x$  (0.010) |
| Male | ARIMA (0,1,0)  (0.015) | $y=0.24-0.014x+0.0005x^{2}$  (0.0093) |
| **Race and ethnicity** | | |
| Non-Hispanic Whites | ARIMA (0,1,0)  (0.015) | $y=0.17-0.0045x$  (0.0095) |
| Non-Hispanic Blacks | ARIMA (0,1,0)  (0.047) | $y=0.60-0.07x+0.003x^{2}$  (0.026) |
| Hispanics | ARIMA (0,1,0)  (0.049) | $y=0.24+0.012x-0.0015x^{2}$  (0.032) |
| Non-Hispanic AI/AN | -- | -- |
| Non-Hispanic Asians | ARIMA (0,1,0)  (0.025) | $y=0.12+0.03x-0.006x^{2}$+0.0002$x^{3}$  (0.017) |
| **Female by age** | | |
| 19-44 years | ARIMA (0,1,0)  (0.008) | $y=0.02+0.009x-0.001x^{2}$+0.00006$x^{3}$  (0.007) |
| 45-64 years | ARIMA (0,1,0)  (0.019) | $y=0.19-0.01x+0.0004x^{2}$  (0.011) |
| ≥65 years | ARIMA (0,1,0)  (0.091) | $y=1.0-0.04x$  (0.061) |
| **Male by age** | | |
| 19-44 years | ARIMA (0,1,0)  (0.014) | $y=0.09-0.009x+0.0005x^{2}$  (0.008) |
| 45-64 years | ARIMA (0,1,0)  (0.031) | $y=0.29-0.016x+0.0008x^{2}$  (0.019) |
| ≥65 years | ARIMA (0,1,0)  (0.085) | $y=1.01-0.042x+0.0007x^{2}$  (0.054) |

**Supplementary Table 3. Model parameter in DKA-related mortality in U.S. adults between 2006-2019.**

|  | 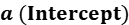  **[95%CI]** | 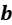  **[95% CI]** | $c$  **[95% CI]** | $d$  **[95% CI]** | 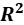^*^ |
| --- | --- | --- | --- | --- | --- |
| **Overall** | | | |  |  |
| Total**^∬^** | 0.973^***^  [0.91 to 1.04] | -0.013  [-0.034 to 0.008] | 0.004^***^  [0.003 to 0.006] | -- | 0.98 |
| **Age** | | | |  |  |
| 0-18 years**^∬^** | 0.060^***^  [0.036 to 0.085] | -0.002  [-0.0096 to 0.0053] | 0.0002  [-0.0003 to 0.0007] | -- | 0.25 |
| 19-44 years**^∭^** | 0.74^***^  [0.66 to 0.82] | -0.068^**^  [-0.11 to 0.023] | 0.011^**^  [0.0041 to 0.018] | -0.0003^*^  [-0.0006 to -0.000035] | 0.97 |
| 45-64 years**^∭^** | 1.26^***^  [1.06 to 1.45] | 0.11^*^  [0.005 to 0.22] | -0.01  [-0.028 to 0.005] | 0.0006**^.^**  [-0.000057 to 0.0014] | 0.97 |
| ≥65 years**^∬^** | 2.72^***^  [2.42 to 3.03] | -0.08**^.^**  [-0.18 to 0.011] | 0.015^***^  [0.0087 to 0.021] | -- | 0.95 |
| **Sex** | | | |  |  |
| Female**^∬^** | 0.81^***^  [0.73 to 0.89] | -0.009  [-0.03 to -0.016] | 0.003^**^  [0.0016 to 0.0049] | -- | 0.96 |
| Male**^∬^** | 1.14^***^  [1.08 to 1.21] | -0.019**^.^**  [-0.038 to 0.0014] | 0.005^***^  [0.0040 to 0.0066] | -- | 0.99 |
| **Race and ethnicity** | | | |  |  |
| Non-Hispanic Whites**^∬^** | 0.82^***^  [0.76 to 0.88] | -0.002  [-0.02 to 0.02] | 0.004^***^  [0.0026 to 0.0049] | -- | 0.76 |
| Non-Hispanic Blacks**^∬^** | 2.29^***^  [0.54 to 0.66] | -0.08^*^  [-0.089 to -0.052] | 0.008^**^  [0.0022 to 0.0046] | -- | 0.99 |
| Hispanics**^∬^** | 1.02^***^  [0.86 to 1.18] | -0.007  [-0.06 to 0.04] | 0.003^*^  [0.0002 to 0.007] | -- | 0.88 |
| Non-Hispanic AI/AN**^∭^** | 2.04^***^  [1.25 to 2.82] | -0.30  [-0.74 to 0.14] | 0.058**^.^**  [-0.009 to 0.13] | -0.003**^.^**  [-0.0055 to 0.0004] | 0.67 |
| Non-Hispanic Asians**^∬^** | 0.61^***^  [0.50 to 0.72] | -0.047^**^  [-0.079 to -0.014] | 0.0048^*^  [0.0027 to 0.0069] | -- | 0.88 |
| **Female by age** | | | |  |  |
| 0-18 years**^∬^** | 0.06^***^  [0.04 to 0.08] | -0.002  [-0.0096 to 0.0052] | 0.0002  [-0.00025 to 0.0007] | -- | 0.25 |
| 19-44 years**^∭^** | 0.57^***^  [0.04 to 0.07] | -0.098^**^  [-0.008 to 0.001] | 0.016^**^  [-0.0001 to 0.0004] | -0.0006^**^  [-0.0001 to 0.0004] | 0.93 |
| 45-64 years**^∭^** | 0.99^***^  [0.74 to 1.25] | 0.10  [-0.04 to 0.24] | -0.011  [-0.033 to 0.010] | 0.0006  [-0.0003 to 0.002] | 0.92 |
| ≥65 years**^∬^** | 2.64^***^  [2.27 to 3.00] | -0.06  [-0.17 to 0.055] | 0.012^**^  [0.005 to 0.019] | -- | 0.92 |
| **Male by age** | | | |  |  |
| 0-18 years**^∫^** | 0.047^***^  [0.028 to 0.066] | 0.0025^*^  [0.0003 to 0.0047] | -- | -- | 0.34 |
| 19-44 years**^∬^** | 0.90^***^  [0.84 to 0.97] | -0.030^**^  [-0.05 to -0.010] | 0.004^***^  [0.003 to 0.006] | -- | 0.97 |
| 45-64 years**^∬^** | 1.68^***^  [1.55 to 1.81] | 0.03  [-0.01 to 0.07] | 0.005^**^  [0.0025 to 0.0078] | -- | 0.98 |
| ≥65 years**^∬^** | 2.83^***^  [2.51 to 3.16] | -0.12^*^  [-0.22 to -0.02] | 0.018^***^  [0.012 to 0.025] | -- | 0.96 |

**Supplementary Table 4. Model parameter in HHS-related mortality in U.S. adults between 2006-2019.**

|  | 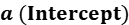  **[95%CI]** | 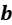  **[95% CI]** | $c$  **[95% CI]** | $d$  **[95% CI]** | 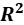^*^ |
| --- | --- | --- | --- | --- | --- |
| **Overall** | | | |  |  |
| Total**^∫^** | 0.21^***^  [0.20 to 0.22] | -0.006^***^  [-0.008 to -0.004] | -- | -- | 0.85 |
| **Age** | | | |  |  |
| 19-44 years**^∬^** | 0.06^***^  [0.04 to 0.07] | -0.003  [-0.008 to 0.001] | 0.0002  [-0.0001 to 0.0004] | -- | 0.31 |
| 45-64 years**^∬^** | 0.24^***^  [0.22 to 0.25] | -0.011  [-0.017 to -0.005] | 0.0005  [0.000098 to 0.00088] | -- | 0.75 |
| ≥65 years**^∫^** | 1.03^***^  [0.97 to 1.09] | -0.040^***^  [-0.043 to -0.030] | -- | -- | 0.91 |
| **Sex** | | | |  |  |
| Female**^∫^** | 0.20^***^  [0.18 to 0.21] | -0.006^***^  [-0.008 to -0.005] | -- | -- | 0.85 |
| Male**^∬^** | 0.24^***^  [0.22 to 0.27] | -0.014^***^  [-0.025 to -0.007] | 0.0005^***^  [-0.00068 to 0.0009] | -- | 0.88 |
| **Race and ethnicity** | | | |  |  |
| Non-Hispanic Whites**^∫^** | 0.17^***^  [0.16 to 0.19] | -0.0045^***^  [-0.006 to -0.003] | -- | -- | 0.76 |
| Non-Hispanic Blacks**^∬^** | 0.60^***^  [0.54 to 0.66] | -0.07^***^  [-0.089 to -0.052] | 0.0034^***^  [0.0022 to 0.0046] | -- | 0.91 |
| Hispanics**^∬^** | 0.24^***^  [0.17 to 0.32] | 0.01  [-0.01 to 0.035] | -0.0015^***^  [-0.003 to -0.00002] | -- | 0.62 |
| Non-Hispanic AI/AN**^∬^** | -- | -- | -- | -- | -- |
| Non-Hispanic Asians**^∭^** | 0.12^***^  [0.059 to 0.19] | 0.03^.^  [-0.0046 to 0.068] | -0.006^*^  [-0.011 to -0.00009] | 0.0002^.^  [-0.00002 to 0.00047] | 0.75 |
| **Female by age** | | | |  |  |
| 19-44 years**^∭^** | 0.02^***^  [0.003 to 0.05] | 0.009  [-0.006 to 0.02] | -0.001  [-0.004 to 0.0008] | 0.00006  [-0.00004 to 0.0002] | 0.14 |
| 45-64 years**^∬^** | 0.19^***^  [0.17 to 0.22] | -0.01^*^  [-0.02 to -0.002] | 0.0004^.^  [-0.00004 to 0.00092] | -- | 0.62 |
| ≥65 years**^∫^** | 1.0^***^  [0.97 to 1.13] | -0.04^***^  [-0.05 to -0.03] | -- | -- | 0.87 |
| **Male by age** | | | |  |  |
| 19-44 years**^∬^** | 0.09^***^  [0.07 to 0.10] | -0.009^***^  [-0.015 to -0.004] | 0.0005  [0.0001 to 0.0008] | -- | 0.64 |
| 45-64 years**^∬^** | 0.29^***^  [0.25 to 0.34] | -0.016^*^  [-0.03 to -0.003] | 0.0008^.^  [-0.00012 to 0.0017] | -- | 0.57 |
| ≥65 years**^∬^** | 1.01^***^  [0.89 to 1.14] | -0.04^*^  [-0.08 to -0.004] | 0.0007  [-0.002 to 0.003] | -- | 0.85 |

^*^
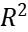
: the goodness of fit, which is used to describe the fitting degree of the regression equation to the observed value. The value range of
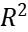
 is [0,1]. The closer
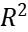
 is to 1, the better the fitting degree is.

**^∫^**
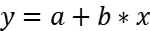


**^∬^**$y=a+b*x+c*x^{2}$

**^∭^**$y=a+b*x+c*x^{2}+d*x^{3}$

**Supplementary Table 5. Characteristics of DKA-related death in the United States, 2006-2021**

|  | **Deaths [%]**  **2006-2021** | **Deaths [%]**  **2006** | **Deaths [%]**  **2019** | **Deaths [%]**  **2020** | **Deaths [%]**  **2021** |
| --- | --- | --- | --- | --- | --- |
| **Overall** | 71,575 [100.0] | 2,811 [100.0] | 5,622 [100.0] | 8,370 [100.0] | 9,650 [100.0] |
| **Age** |  |  |  |  |  |
| 0-18 | 896 [1.3] | 42 [1.5] | 47 [0.9] | 108 [1.3] | 99 [1.0] |
| 19-44 | 15,503 [21.7] | 737 [26.2] | 1,132 [20.1] | 1,802 [21.5] | 1,961 [20.3] |
| 45-64 | 27,788 [38.8] | 1,022 [36.4] | 2,138 [38.0] | 3,001 [35.9] | 3,595 [7.3] |
| ≥65 | 27,388 [38.2] | 1,010 [35.9] | 2,305 [41.0] | 3,459 [41.3] | 3,995 [41.4] |
| **Sex** |  |  |  |  |  |
| Female | 31,491[44.0] | 1,272 [45.3] | 2,468 [43.9] | 3,437 [41.1] | 4,141 [42.9] |
| Male | 40,084[56.0] | 1,539 [54.7] | 3,154 [56.1] | 4,933 [58.9] | 5,509 [57.1] |
| **Race/Ethnicity** |  |  |  |  |  |
| Non-Hispanic Whites | 39,167 [63.3] | 1,764 [62.8] | 3,650 [64.9] | 4,725 [56.4] | NA |
| Non-Hispanic Blacks | 13,212 [21.3] | 691 [24.6] | 1,064 [18.9] | 1,938 [23.2] | NA |
| Hispanics | 7,248 [11.7] | 255 [9.1] | 677 [12.1] | 1,334 [15.9] | NA |
| Non-Hispanic AI/AN | 754 [1.2] | 35 [1.2] | 55 [1.0] | 132 [1.6] | NA |
| Non-Hispanic Asians | 1,544 [2.5] | 66 [2.3] | 176 [3.1] | 241 [2.9] | NA |

AI/AN: American Indian/Alaska Native.

**Supplementary Table 6. Characteristics of HHS - related death in the United States, 2006-2021**

|  | **Deaths [%]**  **2006-2021** | **Deaths [%]**  **2006** | **Deaths [%]**  **2019** | **Deaths [%]**  **2020** | **Deaths [%]**  **2021** |
| --- | --- | --- | --- | --- | --- |
| **Overall** | 8,618 [100.0] | 608 [100.0] | 467 [100.0] | 624 [100.0] | 636 [100.0] |
| **Age** |  |  |  |  |  |
| 0-18 | 68 [0.8] | NA | NA | NA | NA |
| 19-44 | 718 [8.3] | 54 [8.9] | 42 [9.0] | 46 [7.4] | 63 [9.9] |
| 45-64 | 2,563 [29.8] | 167 [27.5] | 147 [31.5] | 207 [33.2] | 193 [30.3] |
| ≥65 | 5,266 [61.1] | 383 [63.0] | 275 [58.9] | 366 [58.7] | 366 [57.5] |
| **Sex** |  |  |  |  |  |
| Female | 4,431 [51.4] | 322 [53.0] | 240 [51.4] | 302 [48.4] | 287 [45.1] |
| Male | 4,187 [48.6] | 286 [47.0] | 227 [48.6] | 322 [51.6] | 349 [54.9] |
| **Race/Ethnicity** |  |  |  |  |  |
| Non-Hispanic Whites | 5,176 [64.8] | 405 [66.6] | 301 [64.5] | 359 [57.5] | NA |
| Non-Hispanic Blacks | 1,541 [19.3] | 134 [22.0] | 104 [22.3] | 148 [23.7] | NA |
| Hispanics | 929 [11.6] | 51 [8.4] | 48 [10.3] | 82 [13.2] | NA |
| Non-Hispanic AI/AN | NA | NA | NA | NA | NA |
| Non-Hispanic Asians | 263 [3.3] | 13 [2.1] | 12 [2.6] | 28 [4.5] | NA |

AI/AN: American Indian/Alaska Native.

**Supplementary Table 7**. **Age-standardized mortality rate and annual percentage change (APC) in DKA-related mortality among U.S. adults, between 2006 and 2020, by race/ethnicity.**

| **Age-Standardized Mortality Rate Per 100,000 Persons** | | | | | | **Temporal Trend Differences** | |
| --- | --- | --- | --- | --- | --- | --- | --- |
|  | Pre-Pandemic  Epoch, 2006 | Pre-Pandemic  Epoch, 2019 | Pandemic Epoch 1  2020 | | | Trend Segments | APC [95% CI] |
| **Race/Ethnicity** | Observed | Observed | Observed | Predicted  [95% CI] | % Difference^†^ |  |  |
| **Non-Hispanic Whites** | 0.81 | 1.53 | 1.95 | 1.63  [1.54 to 1.72] | **19.91** | Trend Segment 1  2006 to 2015  Trend Segment 2  2015 to 2018  Trend Segment 3  2018 to 2020 | 4.1* [3.1 to 5.0]  7.9 [-2.3 to 19.2]  15.4* [4.5 to 27.4] |
| **Non-Hispanic Blacks** | 2.19 | 2.66 | 4.74 | 2.91  [2.52 to 3.30] | **62.89** | Trend Segment 1  2006 to 2018  Trend Segment 2  2018 to 2020 | 1.2 [-0.1 to 2.6]  34.9* [7.9 to 68.6] |
| **Hispanics** | 1.05 | 1.61 | 2.99 | 1.69  [1.46 to 1.92] | **77.00** | Trend Segment 1  2006 to 2018  Trend Segment 2  2018 to 2020 | 3.1* [1.7 to 4.5]  39.1* [10.5 to 75.2] |
| **Non-Hispanic AI/AN** | 1.92 | 2.13 | 5.00 | 1.99  [1.03 to 2.96] | **151.18** | Trend Segment 1  2006 to 2018  Trend Segment 2  2018 to 2020 | 3.3* [0.6 to 6.1]  33.3 [-14.8 to 108.5] |
| **Non-Hispanic Asians** | 0.62 | 0.89 | 1.17 | 0.99  [0.84 to 1.15] | **18.43** | Trend Segment 1  2006 to 2009  Trend Segment 2  2009 to 2020 | -11.7 [-24.8 to 3.6]  8.0* [5.6 to 10.3] |

^†^Denotes % difference between observed and predicted values.

*Indicates that P-value ≤0.05

Non-Hispanic AI/AN, Non-Hispanic American Indian/Alaska Native

**Supplementary Table 8. Age-standardized mortality rate and annual percentage change (APC) in HHS-related mortality among U.S. adults, between 2006 and 2020, by race/ethnicity.**

| **Age-Standardized Mortality Rate Per 100,000 Persons** | | | | | | **Temporal Trend Differences** | |
| --- | --- | --- | --- | --- | --- | --- | --- |
|  | Pre-Pandemic  Epoch, 2006 | Pre-Pandemic  Epoch, 2019 | Pandemic Epoch 1  2020 | | | Trend Segments | APC [95% CI] |
| **Race/Ethnicity** | Observed | Observed | Observed | Predicted  [95% CI] | % Difference^†^ |  |  |
| **Non-Hispanic Whites** | 0.18 | 0.12 | 0.14 | 0.11  [0.08 to 0.13] | **24.06** | Trend Segment 1  2006 to 2020 | -2.6* [-3.7 to -1.5] |
| **Non-Hispanic Blacks** | 0.51 | 0.27 | 0.38 | 0.30  [0.22 to 0.39] | **26.55** | Trend Segment 1  2006 to 2014  Trend Segment 2  2014 to 2020 | -10.3* [-14.2 to -6.2]  6.5 [-0.6 to 14.1] |
| **Hispanics** | 0.25 | 0.12 | 0.21 | 0.09  [0.00 to 0.20] | **133.06** | Trend Segment 1  2006 to 2020 | -4.2* [-6.6 to -1.8] |
| **Non-Hispanic Asians** | 0.13 | 0.07 | 0.14 | 0.09  [0.01 to 0.17] | **51.10** | Trend Segment 1  2006 to 2020 | -5.0* [-7.9 to -2.0] |

^†^Denotes % difference between observed and predicted values.

*Indicates that P-value ≤0.05

The death number for non-Hispanic AI/AN is less than 9.

**Supplementary Table 9. Age-standardized mortality rate and annual percentage change (APC) in DKA-related mortality among U.S. adults, between 2006 and 2020, by race/ethnicity and age.**

| **Age-standardized mortality rate (per 100,000 persons)** | | | | | | **Temporal Trend Differences** | |
| --- | --- | --- | --- | --- | --- | --- | --- |
|  | 2006 (Pre-Pandemic Referent Epoch) | Pre-Pandemic  Epoch, 2019 | 2020 (Pandemic Epoch 1) | | | Trend Segments | APC [95% CI] |
|  |  |  | Observed | Predicted [95%CI] | % Increase ^†^ |  |  |
| **Non-Hispanic Whites** | | | | | | | |
| 19-44 years | 0.58 | 1.03 | 1.35 | 1.08  [1.00 to 1.16] | **25.03** | 2006-2010  2010-2020 | -0.3 [-5.6 to 5.3]  7.6* [-6.1 to 9.0] |
| 45-64 years | 1.12 | 2.39 | 2.95 | 2.59  [2.40 to 2.77] | **13.85** | 2006-2018  2018-2020 | 5.1* [4.3 to 5.8]  17.8* [3.9 to 33.4] |
| ≥65 years | 2.30 | 3.95 | 5.19 | 4.38  [4.11 to 4.66] | **18.45** | 2006-2015  2015-2020 | 2.8* [1.3 to 4.5]  12.0* [7.9 to 16.4] |
| **Non-Hispanic Blacks** | | | | | | | |
| 19-44 years | 1.71 | 1.90 | 3.52 | 1.97  [1.68 to 2.27] | **78.75** | 2006-2012  2012-2020 | -4.9 [-12.0 to 2.9]  10.3* [4.9 to 16.0] |
| 45-64 years | 3.36 | 4.34 | 7.40 | 4.61  [4.14 to 5.09] | **60.45** | 2006-2018  2018-2020 | 1.4* [0.3 to 2.6]  33.3* [9.7 to 62.0] |
| ≥65 years | 5.16 | 6.28 | 11.33 | 6.16  [5.44 to 6.89] | **83.98** | 2006-2018  2018-2020 | 0.7 [-1.3 to 2.7]  33.3 [-4.9 to 86.7] |
| **Hispanics** | | | | | | | |
| 19-44 years | 0.36 | 0.54 | 1.26 | 0.57  [0.52 to 0.62] | **120.88** | 2006-2018  2018-2020 | 2.0* [0.3 to 3.8]  42.5* [6.1 to 91.5] |
| 45-64 years | 1.23 | 2.19 | 3.81 | 2.09  [1.94 to 2.25] | **82.23** | 2006-2018  2018-2020 | 3.1* [1.7 to 4.5]  35.8* [7.4 to 71.6] |
| ≥65 years | 4.51 | 6.37 | 11.57 | 6.92  [5.75-8.09] | **67.15** | 2006-2018  2018-2020 | 3.6* [0.4 to 6.8]  40.1 [-16.8 to 136.2] |

^†^Denotes % difference between observed and predicted values.

*Indicates that P-value ≤0.05

**Supplementary Fig.1. Observed age-standardized mortality rates for DKA and HHS-related deaths before and during the COVID-19 pandemic by race/ethnicity.** (A) DKA-related, (B) HHS-related.


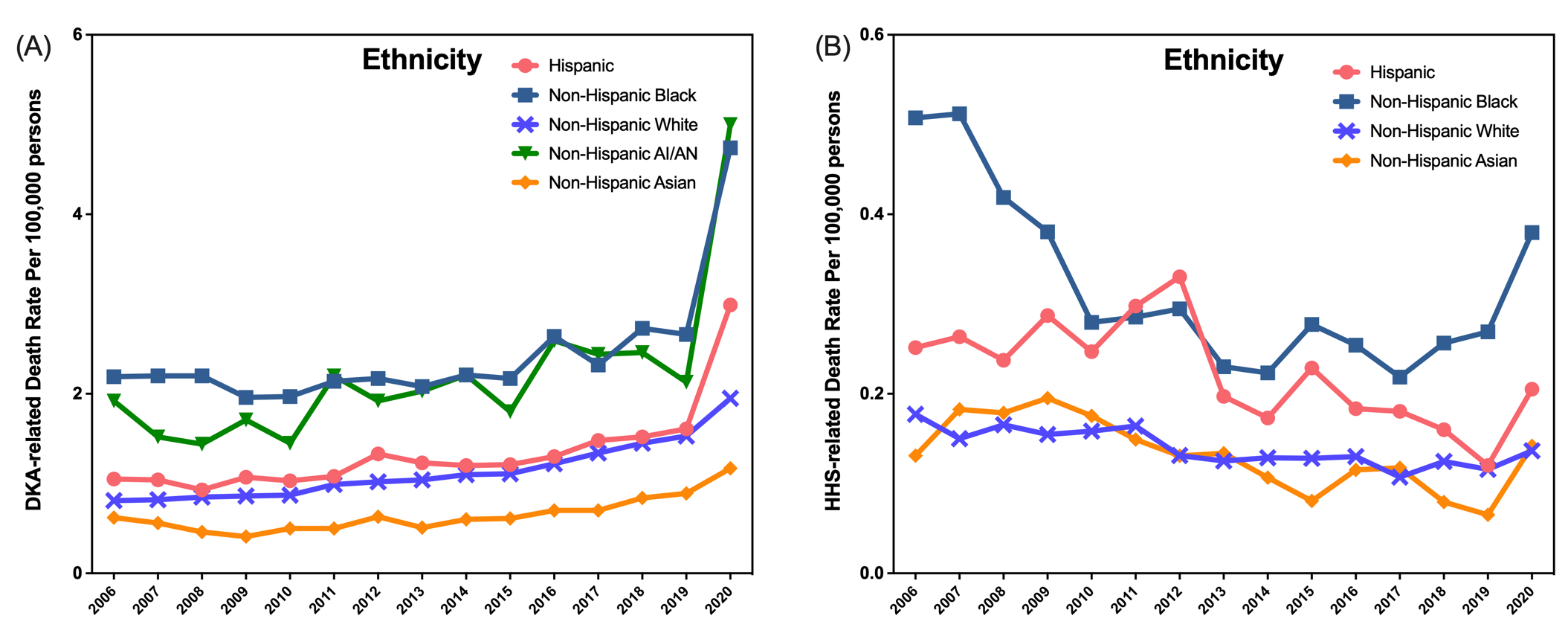

Supplement: Supplementary file 1 — Data S1. Supporting information. [file JDB-16-e13591-s001.docx]
